# Building on the EGIPPS performance assessment: the multipolar framework as a heuristic to tackle the complexity of performance of public service oriented health care organisations

**DOI:** 10.1186/1471-2458-14-378

**Published:** 2014-04-17

**Authors:** Bruno Marchal, Tom Hoerée, Valéria Campos da Silveira, Sara Van Belle, Nuggehalli S Prashanth, Guy Kegels

**Affiliations:** 1 Department of Public Health, Institute of Tropical Medicine, Antwerp, Belgium; 2 Politics and Policy Group, Faculty of Public Health and Policy, London School of Hygiene and Tropical Medicine, London, UK; 3 Institute of Public Health, Bangalore, India

## Abstract

**Background:**

Performance of health care systems is a key concern of policy makers and health service managers all over the world. It is also a major challenge, given its multidimensional nature that easily leads to conceptual and methodological confusion. This is reflected by a scarcity of models that comprehensively analyse health system performance.

**Discussion:**

In health, one of the most comprehensive performance frameworks was developed by the team of Leggat and Sicotte. Their framework integrates 4 key organisational functions (goal attainment, production, adaptation to the environment, and values and culture) and the tensions between these functions.

We modified this framework to better fit the assessment of the performance of health organisations in the public service domain and propose an analytical strategy that takes it into the social complexity of health organisations. The resulting multipolar performance framework (MPF) is a meta-framework that facilitates the analysis of the relations and interactions between the multiple actors that influence the performance of health organisations.

**Summary:**

Using the MPF in a dynamic reiterative mode not only helps managers to identify the bottlenecks that hamper performance, but also the unintended effects and feedback loops that emerge. Similarly, it helps policymakers and programme managers at central level to better anticipate the potential results and side effects of and required conditions for health policies and programmes and to steer their implementation accordingly.

## Introduction

Performance of health care systems is a key concern of policy makers and health service managers all over the world. Demands for better quality of care, higher productivity, better responsiveness, more efficiency and better sustainability are all expressions of the same question of how to improve performance of health services and health workers [1,2]. In the health sector, performance remains a difficult issue because of its multidimensional nature [3]. This easily leads to conceptual and methodological confusion and is reflected by a scarcity of models to analyse the performance at health system level [4,5].

Not surprisingly, virtually all current frameworks include quality of care as a key element [6]. Also effectiveness, productivity and efficiency are recurrent themes, for instance in the World Health Report 2000 [7] and the OECD framework [8]. In contrast, social outcomes of health care and equity are missing or little developed in most frameworks, with Australian and Canadian national frameworks as notable exceptions [4]. Furthermore, to our knowledge, only the frameworks developed by Priester [9] and Handler and colleagues [10] and the Dynamic Health System framework [11] explicitly mention values and organisational culture as a key element of performance.

In the health sector, one framework stands out in this crowd: the framework developed by Sicotte et al. [12]. On the basis of a literature review by Leggat and colleagues [13], Sicotte and colleagues developed a comprehensive framework for the assessment of performance of health care organisations. Theirs is a framework of performance that includes goal attainment, production and adaptation to the environment as core dimensions of performance, but it usefully adds a focus on values and culture. The Sicotte framework is geared towards North American settings and has been mainly used in OECD countries, for instance as the basis of WHO-Europe’s framework for assessment of hospitals [1], to assess accreditation schemes [6], to analyse how actors and stakeholders of a health care organisations define performance [14-17] and to explore how health care organisations learn [18]. In Francophone and Lusophone countries, the framework goes by the name of ‘le modèle d’Evaluation Globale et Intégrée de la Performance des Systèmes de Santé’ or the acronym EGIPSS [19,20]. We use this acronym in this paper as shorthand for the framework developed by Sicotte and colleagues.

In this paper, we present the multipolar performance framework (MPF). Keeping the key strengths of the Sicotte framework, we redefined some elements on the basis of concepts of integrated health systems and public service. We also adapted the framework to facilitate the analysis of the relations and interactions between the multiple actors that make health organisations complex. Since most performance frameworks can be considered to be either structuralist or functionalist in nature, we argue that the relational perspective of the MPF makes it more suitable to deal with the social complexity of health organisations. This allows indeed for an analytical strategy to understand the organisational dynamics. Finally, it is the lean nature of the MPF that makes it so effective: more than a structured set of indicators for each function, the MPF calls attention to the dynamic linkages between these functions. The MPF is indeed best considered as a meta-frame or a heuristic that can help managers, policymakers and researchers alike to make sense of performance of any health organisation.

The paper starts with the key features of the EGIPPS framework. We then present the multipolar performance framework and illustrate how it can be used to assess the performance of health organisations. In the discussion section, we present the limitations of the MPF, its use as a meta-frame of analysis and its added value compared with other frameworks.

## Background

**Figure 1:**
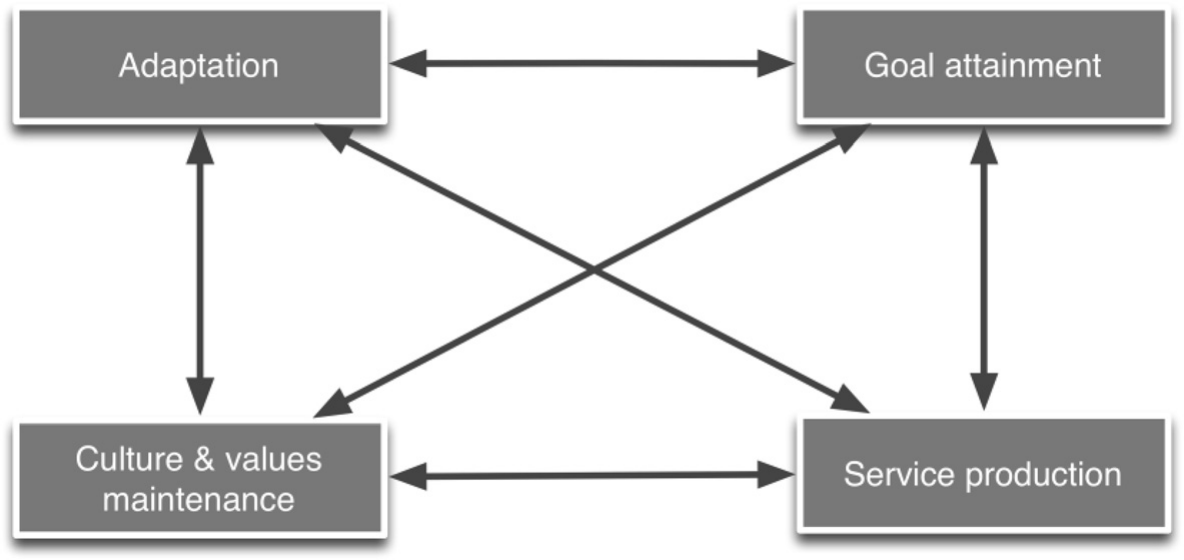
**The four functions of the EGIPPS framework of Sicotte et al. **[12]**.**

The work of the team of Sicotte and colleagues is based on a literature review that showed that all existing frameworks to assess the performance of health care organisations (HCO) were missing important dimensions [13]. The team found inspiration in Parsons’ social system action theory [21] and the competing values framework of Quinn and Rohrbaugh to develop an integrative framework of performance [22]. Sicotte et al. consider that performance of a HCO is multi-dimensional. More specifically, it is the result of the interaction between four organisational functions: production, goal setting, values & culture maintenance, and adaptation to the environment (Figure 1). The success of an organisation depends not only on how each of these functions is organised, but also on how they are aligned with each other. Performance is therefore understood as more comprehensive than merely efficiently producing desired outputs [12].

### Key features

#### Goal attainment

Any health care organisation has aims and goals it wants to achieve. Goals that are clear and specific and that are shared by all members are powerful ‘pull’ factors that can direct both staff and organisation. Goal setting is therefore a key responsibility of management. Sicotte et al. define the key goals of HCO as including effectiveness, efficiency and stakeholder satisfaction.

#### Service production function

In order to reach its goals, a HCO needs to organise and coordinate its internal production processes, which consist of clinical and support services. Traditionally, evaluations of HCO performance focus mainly on this function, assessing it in terms of volume, cost and quality of services. Sicotte et al. add productivity and coordination of production factors as elements.

#### The adaptation function

Health care organisations need to interact with their environment to obtain manpower, financial resources, drugs and equipment. They also draw non-tangible resources from their environment: respect, authority, trust, reputation, knowledge, etc. However, the relationship with the environment is bidirectional. HCOs are expected to respond to the needs and priorities of the population and other stakeholders, and to take their respective values into account. Sicotte at al. therefore include market presence and capacity for learning and innovation as elements of the adaptation function.

#### Culture and values maintaining function

Sicotte et al. subscribe to Parsons’ view that all human action is ultimately generated by the values–hidden and implicit or open and known–of the actors. Maintaining values contributes to good relations between the people working in the organisation and thus to cohesion within the organisation. Parsons called this the pattern maintaining function and considered it to be the most important function in human organisations [23]. For Sicotte and colleagues, this function consists of maintaining the fundamental values – in HCO often dominated by professional values like patient dedication, ethics and professional autonomy – and the organisational climate, which in HCOs is supposed to be geared towards collaboration.

#### The alignments

**Figure 2:**
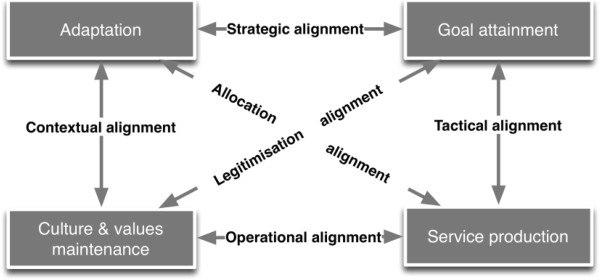
**The alignments in the framework of Sicotte et al. **[12]**.**

The framework describes six alignments between these four functions, which can be best understood as tensions that may arise between functions as a result of a change in one of them (Figure 2). The tactical alignment links the *Goal attainment* and *Service production* function. This deals first with the appropriateness of the service provision in relation to the goals: “To what extent do the service production processes contribute to attaining the goals? Are they effectively producing the output needed to reach the goals?”. Second, this alignment relates goals to the service provision capacity: “Are the chosen goals within reach of the organisation given its delivery capacity?”.

The allocation alignment links the *Interaction with the environment* and the *Service production* function. It first deals with resource acquisition. Questions that can be used to assess this include: “Are the obtained resources adequate to organise the service production function? Is the service production function optimal in relation to available resources?”. It also covers the opposite direction, i.e. the issue of responsiveness: “Are the right services provided for the population for which the HCO is responsible? Are the services acceptable to the population? Are all relevant stakeholders taken into account when setting service delivery priorities?”.

The strategic alignment examines the link between the *Goals* that the HCO is pursuing and its *Environment*. Here, questions include whether the organisational goals correspond with the needs of the population and other key actors. Inversely, one assesses the influence of external actors on organisational goal setting: “Who influences the goals and how? How is the alignment between the external actors and the goals of the HCO?”.

The legitimisation alignment is about the congruence of the *Goal attainment* function with the *Culture and values maintaining* function, and inversely questions how the strategic choice of goals influences and shapes the organisational values.

The operational alignment covers the congruence of the *Culture and values maintaining* function with the *Service production* modalities, and the impact of the service production system on the organisational culture and values.

Finally, the contextual alignment between *Culture and values maintaining* function and *Adaptation to the environment* deals with how the social, political and cultural dimensions of the environment influence the organisational culture and its core operational values, and inversely, whether and how the organisational culture is congruent (or not) with the environment of the HCO.

With this framework, the assessment of the performance of a health care organisation therefore covers four functions and six alignments.

### Strengths and challenges

Whereas previous performance frameworks focused on some of the four functions or favoured particular management theories, Sicotte et al. integrated the main streams of management into one comprehensive performance framework, usefully pushing the definition of performance beyond the assessment of how service provision contributes to goal attainment.

As mentioned in the Introduction, the EGIPPS framework was applied mainly in OECD countries. Our citation analysis of the papers that refer to the publication of Sicotte et al. shows that the framework is indeed often cited in support to the argument of the multi-dimensional or elusive nature of performance [24-30], of the subsequent need to understand the definition of performance by the various stakeholders of the HCO [15,31-33] and of the need to adapt management approaches to this complex nature of performance of HCO [34]. It inspired other authors in the development of comprehensive literature reviews [35] or conceptual frameworks [36].

The EGIPPS framework has additional strengths. The interaction with the environment is considered to be bi-directional and to include responsiveness to key stakeholders. Particularly interesting for management of public-oriented health organisations is the explicit place in the framework for the role of values as the driving force of a HCO.

However, the framework presents some challenges. First, going from a traditional assessment of two functions that are assumed to be linearly connected (service production to goal attainment) to four functions and their linkages complicates the assessment of performance. This forces evaluators to assess two additional and largely intangible issues: (1) values, responsiveness and organisational culture, and (2) the alignments. This additional difficulty may explain why relatively few authors use the full framework for actual research. One of the major applications of the framework is the development of the performance assessment tool for quality improvement in hospitals (PATH) by Veillard and colleagues [1]. The 6 dimensions of hospital performance that were withheld include clinical effectiveness, safety, patient centredness, production efficiency, staff orientation and responsive governance. They express a natural focus on the Service Production function of the EGIPPS framework. Also Bittencourt and Hortale, who applied the framework to the analysis of waiting time and overcrowding of hospitals, mainly focused on the technical effectiveness of service production [37].

This sparse use of the framework to assess HCO performance in its full spectrum seems to indicate that the assessment of the functions, let alone of the alignments between the functions, can be quite difficult. The analysis of 5 major accreditation manuals by Smits on the basis of the functions of the EGIPPS framework [6] similarly found that just one of them – the Australian guide [38] – focused on balancing alignments. This difficulty is, of course, an issue with all frameworks that embrace a definition of performance that goes beyond production of services.

Second, the EGIPPS framework may easily focus the analyst’s attention to organisational functions and structural alignments, whereas much of the problems underlying organisational performance are related to social interactions and relations. The authors acknowledge the tensions that are likely to arise as a consequence of conflicting interests and the difficulties in arbitrating between conflicting values. They refer to Habermas’ constructive mediation [39] as an approach to establish rules for participation and priority setting, but their definition of the organisational functions does little to acknowledge the social complexity of HCO and to help managers to make sense of this complexity.

### The multipolar performance framework

Confronted by challenges in assessing performance of HCO in low- and middle-income countries and in teaching strategic management to health professionals from these countries, we modified the Sicotte framework in three ways. First, we expanded its scope to include not only health care organisations (HCO), but also health support organisations (HSO), which in most health systems in the South, play an important role. We define an HSO as any organisation that mainly supports care and/or service delivery. Examples include NGOs providing technical and financial support to local districts or hospitals, but also central, regional or provincial health authorities or funding agencies are HSO. This differentiation leads to new categories to describe, for instance, the service production function (see Table 1 for examples). From here on, we will use ‘health organisation’ (HO) to include both HCO and HSO.

**Table 1:** Examples of components of the service delivery function in a range of HOs

	**Hospital**	**First line system**	**Disease control programme**	**NGO supporting districts**	**NGO supporting other NGOs**
**Actual service provision**	Provision of hospital-level diagnosis and care	Health promotion, prevention and curative care	Vector control	Training district management teams	Training NGO managers
			Environmental control	Providing supplies and drugs	Providing material support
			Information, Education and Communication	Provision of targeted services	
			Curative services		
**Operational management**	Management of wards	Team management	Programme management	Coordination with general services	Coordination of own staff
	Procurement Pharmacy Quality management Health information system	Supplies	Financial management coordination with general services	Staff recruitment and training	Communication with the NGOs that are supported
	Continued professional development	Training	Procurement and supply Quality control	Monitoring & evaluation	Supervision of own staff
		Supervision	Health information system	Supervision	Monitoring and evaluation
		Quality management			
		Health information system			

**Figure 3:**
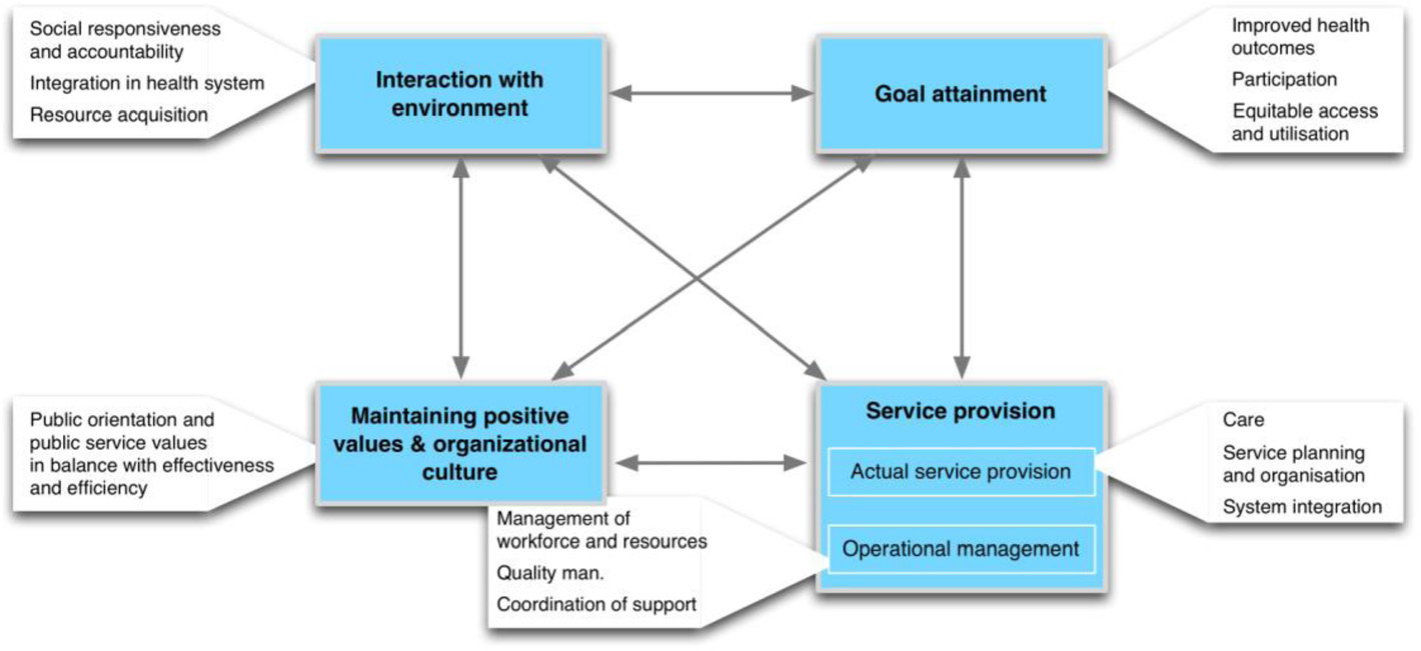
The Multipolar performance framework.

Second, we infused key elements and concepts of integrated health systems and public service in the definition of the sub-dimensions [11,40]. Health organisations operating in the public domain are not value-neutral [5], and neither are health system frameworks. We therefore make explicit the values that influence our definition of the goals that a health organisation should pursue (see Figure 3). The goal of a HO is not (only) maximising efficiency and profit. As social institutions with a public service perspective, they are intended to provide care and services that contribute to equitable access and utilisation of health services. In the process, they are to be accountable to the communities they serve, and not only to powerful stakeholders [41-43]. The pursuit of such values and objectives affects not only all functions of a HO and its management, but also the reference frame used to evaluate such organisations. To this end, we changed the labels of three of the four functions. We replaced ‘Culture and values maintaining’ to ‘Safeguarding organisational culture and values’ to stress the need of ensuring that the organisational culture promotes positive values. The label ‘Production’ is replaced by ‘Service provision’ and ‘Adaptation to the environment’ by ‘Interaction with the environment’. The latter change emphasises the need for HO managers to actively engage with their key stakeholders and respond to their expectations, instead of undergoing the environmental pressures.

The third modification is an attempt to upgrade the EGIPPS framework to better deal with complexity. The advantage of the EGIPPS framework, much the same as the competing values framework of Quinn and Rohrbaugh [22] did, was to integrate all main schools of management. Since then, however, complexity theory has entered much more strongly into organisational and management theory. Interesting insights were developed in the domain of decision-making [44,45], strategic management [46,47] and leadership [48,49]. One key aspect of complexity theory is the central role of human agency and relations in emergence of change within organisations. The alignments in the EGIPPS framework represent the interaction between the functions and allude to the tensions that often arise as a consequence. However, adopting a functionalist approach, Sicotte and colleagues provide little explanation on how these tensions come about and little guidance to the analysis of these tensions. This modification is, therefore, an analytical strategy to focus attention on the social dynamics within the HO and in its relation with the environment, which accounts for the dynamic interactions within and between functions and the resulting emergence of change, feedback loops and unintended effects. As such, the MPF becomes a heuristic that can help at making sense of complex organisational behaviour.

### Key features

#### Goal attainment

From an organisational theory point of view, institutional survival, and the concomitant concern for efficiency, cost-containment and user satisfaction, is a major driver of any kind of service organisation. The mission of health care organisations, however, includes other goals, most often summarised as “to contribute to better health status and/or well-being of the population”.

Inspired by Groupe d'étude pour une réforme de la medicine [50] and Giusti et al. [41], we argue that the mission of health organisations that are oriented towards public service (in short public HO) includes improving the health status and well-being of the population, supporting the autonomy of individuals and communities and contributing to social justice. Such a mission can be translated in the following goals:

Improved health outcomes of the population of responsibility

Participation of patients, community and other legitimate stakeholders in decisions regarding individual care, community-level action and organisation of health services

Equitable access to and utilisation of quality health care according to need.

In line with the idea that goals need to match the mission, and that the mission is influenced by multiple actors and interests, dialogue among and coordination of the various stakeholders in defining the mission, setting the priority goals and developing the organisational strategy is of key importance. This makes the legitimisation alignment the key axis of the MPF.

#### Service provision

We change ‘service production’ of Sicotte’s framework into ‘service provision’, and distinguish two sub-functions: (1) the actual service provision and (2) the operational management.

The components within the actual service delivery function – and their relative weight – vary in function of the type of HO and its core activities (see Table 1 for examples). *Health care organisations* focus essentially on providing health care and services. For instance, the core activities of a hospital include specialised diagnosis and treatment. *Health support organisations* in essence provide services that enable other organisations to perform better: they typically support the operational management of their target organisation. An NGO supporting a hospital, for instance, may focus its service delivery on training, supply of inputs, etc. For all HOs, the actual service provision can be assessed in terms of quantity, quality and cost of services.

The actual service delivery sub-function includes Care, Service planning and organisation and System coordination. Within the operational management sub-function, we include Management of workforce and resources (finances, knowledge and know-how, material and supplies, infrastructure), Quality management and Coordination of support.

#### Interaction with the environment

The interaction function embodies a bi-directional relationship that focuses on resource acquisition as well as on responsiveness.

In line with a public service value set, (social or community) responsiveness means in essence to respond to population needs, to health system demands and to relevant societal and political influences. Changing demands and needs should lead to reviewing present service delivery and to modification if needed. HO management teams also need to be accountable to the legitimate stakeholders. In this light, the interaction can be assessed by looking at the voice and power given to patients and communities in matters of goal setting and management of the HO.

An element we add to the EGIPPS framework is the role of the HO in the wider health system. This refers to the notion of integrated health systems [51,52] and includes maintaining effective linkages with other tiers of the health system (including, for instance, participating in patient referral systems or providing training and supervision).

#### Safeguarding the organisational culture and values

The organisational culture consists of the behaviours, artefacts and norms that prescribe and sanction the behaviour of organisational members. This visible layer is informed by the values that influence the behaviour of staff and their beliefs and assumptions [53,54]. The multiple groups of actors in health organisations shape the organisational culture and create their own subculture. In a HO, professional values (both medical and public health values), bureaucratic norms and institutional survival mix with staff members’ personal values. Finally, the organisational culture is influenced by the societal values. The interactions between actors in and outside of the organisation lead to “*some measure of dependable coordinated behaviour*” [49], mainly through developing (or not) shared value sets. In other words, the organisational culture is both a driver of social complexity as well as the result of it.

#### The alignments between the functions

Assessing the alignment between 2 functions exposes the coherence between these functions. This was one of the major innovations of the EGIPPS framework, as it allows for a systemic analysis of performance. With the MPF, we push the analysis of the alignments into an analytical strategy to understand the organisational dynamics. To this end, the perspectives of actors inside and outside the organisation and their relations are explored, specifically in regards to the one alignment that is *primus inter pares*: the legitimisation alignment between *Safeguarding the organisational culture and values* and *Goal attainment*. In-depth stakeholder analysis [55,56] or an assessment of the power relations [57] are methods to assess how priorities and objectives are defined and by whom.

### Dynamic assessments of performance

The most straightforward use of the multipolar framework is to provide a *static description* of the organisational performance in its four key functions–a snapshot of the current performance. The paper by Sicotte et al. presents some evaluation questions and indicators to this end, and indicators from other frameworks can be transposed to the corresponding elements of the MPF. In its most basic application, the MPF remains close to the EGIPPS framework and is indeed best considered as a neutral meta-frame. Such static description can be used to compare the current performance with the objectives of the strategic plan, the national norms or the performance of other HOs.

However, the real power of the MPF lies in its capacity to facilitate a *dynamic assessment* on the basis of the alignments. This use of the MPF as a heuristic is in line with a complex systems approach to organisations and the assessment of their performance [58].

A dynamic assessment of performance starts with the triangle *Goal attainment–Service provision–Interaction with the environment*. This is the common approach to evaluation of performance, in which case the cause of the inadequate goal attainment is sought, first, within the service delivery function, second, in the operational management capacity, and third, in the acquisition of resources and recruitment of manpower. If this first stage does not explain the present performance gaps, the feasibility of the goals needs examination (the tactical alignment). Goals won’t be attained if they are set too high relative to the organisational capacity.

However, the MPF goes further than a traditional performance assessment. The second phase concerns the triangle *Goal attainment–Culture and values–Interaction with the environment.* The relevance of the goals relates to the legitimisation alignment. Here, a first question is in how far the goals are coherent with the mission of the HO: organisations pursuing goals that are not supported by a shared vision and mission often fail to mobilise their personnel. Second, the goals also need to be relevant for the legitimate key actors, among whom the patients, their families and communities – this deals with responsiveness and the strategic alignment.

This then leads to the third phase: the assessment of the influence of the *external actors* on the HO. First, external actors often have an influence on goal setting, for instance by imposing performance objectives or policy goals. Second, they shape the service provision through (earmarked) resources, knowledge and other means (the allocation alignment). Through funding particular activities or providing specific targeted supplies, for instance, external actors shape the service provision capacity. Strategic use of stakeholder analysis and power analysis can help to map the key actors, their power and influence, their interests (in the HO), and not in the least, their legitimacy.

In a fourth phase, a dynamic analysis focuses on the formal and informal *values* that are maintained by the HO and on how these values actually shape the mission statement and the goals, as well as the actual service delivery. Key questions include: “Who shapes the organisational culture? Through which structures, relations and processes are the organisational values expressed and operationalized? Whom does the culture serve? What are the power relations and differentials it maintains and by which it is maintained?”.

The above sequence of 4 phases is best run *reiteratively:* any change in one function and the management or staff’s response to it has the potential to influence other functions through the alignments. Such reiterative analysis often leads to a better understanding of the evolution of the HO and how and why its performance changed in time. This means that the emphasis shifts from merely describing the current state of each function and its elements on the basis of quantitative indicators to an analysis of the interactions between an event or change in one function and the resulting changes in other functions. It necessarily calls for exploring the functions of *Interaction with the environment* and *Culture and values* and their alignments*,* which is hardly done in the current applications of the EGIPPS framework, and indeed in any performance assessment tool. Such analysis of dynamics can by its very nature not be formalised in a fixed ‘7 step’ procedure, but the above described phases indicate how the reiterative analysis process can be structured.

Below, we present the analysis of the effects of the start of a national health insurance scheme on hospital performance as an example of how the MPF can be applied in a dynamic performance assessment. Instituted over the last decade in a number of countries, including Ghana [59,60], such policies represent a major event for health care organisations. We take a hospital as the unit of analysis, to show how the MPF can help a management team or researchers to anticipate and trace the effects of the policy.

• Aiming at reducing the financial barriers to care, the start of a national health insurance scheme will not change the goals of the hospital. This policy is assumed to lead to better access and utilisation of the hospital by all groups of the population, and especially by the poorest. However, research shows this assumption does hold only if other conditions are met. The effect on the goal attainment needs therefore to be monitored and the health information system of the hospital needs to be adapted if it is not yet documenting utilisation in terms of equity.

• By reducing financial barriers to access, the policy may lead to a rapid increase in the volume of patients. If no preparations are made, the workload and the waiting times are likely to increase. This may have two consequences. First, sudden increases of workload may swamp the existing capacity, put pressure on the quality of care and impact upon staff motivation. Second, reduced staff motivation, combined with long waiting times may colour the patient experience negatively and deter them from using the hospital’s services. This is a negative feedback loop that may reduce future utilisation.

• At the same time, the health insurance policy acts directly upon the allocation alignment: government funding shifts from recurrent funding modalities to subsidising the health insurance fund. This typically affects the revenue of the hospital. In Ghana, for instance, revenue in most hospitals increased significantly due increased utilisation rates, but only in hospitals where the management team prepared well for the new claim processing tasks (positive feedback loop). The operational management capacity proved to be a major potential bottleneck, as delayed or inadequate claim processing directly affects the cash flow. Pro-active teams were seen to institute measures to stimulate their patients to register [61].

• Effective management teams will use the increased financial means to improve capacity and working conditions and thus initiate positive feedback loops. Investing in administrative capacity further enhances the efficiency of the financial management. Increased staff motivation combined with better patient facilities may attract more patients, especially important in settings where patients have alternative options. This was observed in one regional hospital in Ghana, while a comparable hospital missed the opportunity with a stagnation of utilisation rates and a decrease of staff motivation [62].

• The dynamic assessment indeed also points to changes in the hospital’s environment. If the policy covers both private and public actors, it induces competition, not only between public facilities, but also with private facilities: under the new policy all become financially accessible for all adherents. A perceived need to compete with private HCOs may induce improvements in service delivery and may strengthen client responsiveness as an organisational value. Negative goal displacement can occur as well, especially when hospital management teams game the policy and focus on the most profitable services to maximise reimbursements.

• This in turn will affect the service actual delivery, by focusing the attention of health workers to the more lucrative services to the detriment of other responsibilities. It may affect the organisational values and lead to crowding out of professional pro-patient values by the value of profit maximisation.

• The introduction of health insurance may affect some power relations within and around a hospital and not others. For instance, if effective claim processing is essential for keeping the cash flow going, health information clerks and administrative staff gain in bargaining power. This process was observed in an urban district hospital [61]. The policy may empower patients by increasing their choice and allow for effective exit, but only if alternative accredited hospitals are nearby and accessible. The local health insurance office may become a lynchpin in the system, as well as the national agency that sets the reimbursement rates.

This simple example shows how managers can anticipate the potential impact of a policy by using the MPF. It also shows how an evaluation of such a policy that is limited to assessing patient volumes and hospital revenue would be insufficient. To be effective, the policy should increase the actual utilisation of the hospital by the poorest, avoid harming existing services and ensure that no other perverse effects occur.

## Discussion

We explained how the MPF is based on the work by Sicotte et al. and how we modified it in 3 ways: we expanded its scope to include health care and health support organisations, we infused it with key elements of integrated health systems and public service values, and we showed how it can be used to do dynamic performance assessments that take into account the complex nature of health organisations.

We have been using the MPF in the Strategic Management of Health Systems course of the Institute of Tropical Medicine (Antwerp) since 2007. We found it most useful if used as a dynamic framework, in which case it opens up (analytical) perspectives. The MPF, indeed, gives the management team of a HO a helicopter view of their organisation, from which they can oversee its four functions and the alignments. This helps managers to broaden their definition of organisational performance and to move from monitoring of functions through measurement to assessing and managing the social relations and the dynamics of their organisation in its environment.

We argue that a major advantage of the MPF is its parsimonious character. Within this meta-framework, different theories and concepts can be mobilised to understand and explain observed patterns. Its *meta* nature may also be a weakness, as it requires a good knowledge of theories from sociology, psychology, management, organisational studies, etc., and thus ideally a multi-disciplinary team to use it to its fullest potential.

As we mentioned above, one of the challenges of the EGIPPS framework was that it broadened the definition of performance and the subsequent need to assess intangible dimensions of health care. Evidently, the MPF only complicates matters, as it emphasises the (social) complexity of HO and calls attention to the analysis of relations, interests and power. Also the attribution of change in any function to an intervention or event in other functions is difficult: the alignments interconnecting the functions show pathways of multiple determination and feedback. However, if health organisations are complex organisations, we need to embrace complexity and accept that the best we can do is to search for and provide plausible explanations and look for contribution rather than attribution. We found that running the dynamic assessment in a reiterative mode usually helps in developing such plausible explanations.

The MPF differs from the EGIPPS framework by the central place of the organisational culture and values function and the Legitimisation alignment. This is in line with the importance given to organisational culture in the discipline of management, even if in health care, a straightforward link between organisational culture and performance may be difficult to demonstrate [63]. The organisational culture and values shape the goals of the organisation through the legitimisation alignment and influence the provision function to a large extent. However, organisational culture is seldom neutral. Shared values emerge out of relations between the people in an organisation and shape the organisational behaviour. An important distinction is to be made between officially espoused norms and values, and the operational values that underlie actual individual and organisational behaviour. Positive espoused values described in mission statements (e.g. equity, participation, trust) can be easily undermined by negative operational values maintained by demotivated and ill-paid staff or managers. Inversely, the potential negative influence of certain actors or policies may be averted if staff members are strongly motivated by professional or public service values. Examining the organisational culture and its influence on the other functions is therefore a key competence, for managers and researchers alike.

As for any organisation, health organisations represent political arenas in which different groups and cadres form alliances to advance their goals [14]. This reflects the notion of negotiated order, in which the various actors and stakeholders reach a certain equilibrium by means of power struggles, conflicts, negotiation and discussion [64]. Quinn & Rohrbaugh refer to this when they say their competing values framework leads to a “*conflictual, process-oriented, or dialectic view of the nature of organisations*” [22], in which the arrangements to deal with the tensions can be antagonistic. As we saw above, Sicotte et al. acknowledged this and called for a multiple stakeholder-approach to manage the conflicting values pursued by different actors. This is indeed a second key competence of HO managers. The ‘accountability for reasonableness’ framework is a relevant alternative approach to deal with these tensions. It argues that if it is often impossible to choose between priorities on the basis of technical criteria, at least the process of priority-setting can be made more fair. Criteria of fair decision-making practice include democratic decision-making, openness about agenda setting, transparency (providing honest and understandable information), critical reflection, open debate and contestability of decisions [65]. This was later condensed to the four principles of relevance, publicity, appeal and leadership [65-68].

## Summary

The multipolar performance framework builds upon the work by Sicotte et al. [12]. Our modifications include adding concepts of integrated health systems and public-oriented health service organisation. We also adapted the framework to facilitate the analysis of the relations and interactions between the multiple actors that make health organisations complex.

Using the MPF in a dynamic reiterative mode not only facilitates identifying the bottlenecks that hamper performance, but also the unintended effects and feedback loops that emerge. In this sense, it helps managers in understanding the complex nature of their organisation and anticipating unintended effects and making informed strategic choices for improvement.

We argued that the EGIPPS framework may focus the attention to functions and structural alignments, whereas much of the problems underlying organisational performance are related to social interactions and relations. For this reason, we stressed the relational perspective of the MPF that makes it more suitable to deal with the social complexity of health organisations. This includes the central place of decision-making and priority setting, and the role of values in such processes. This pushes performance assessment far beyond the effectiveness and efficiency questions, making it more difficult, but also more relevant.

## Competing interests

The authors declare that they have no competing interests.

## Authors’ contributions

BM, TH and GK contributed to the conception and design of the paper. BM, TH, VcDS, SVB, NSP and GK were involved in drafting of the manuscript and its revisions. BM edited the final draft. All authors read and approved the final manuscript. All authors agree to be accountable for all aspects of the work in ensuring that questions related to the accuracy or integrity of any part of the work are appropriately investigated and resolved.

## Pre-publication history

The pre-publication history for this paper can be accessed here:

http://www.biomedcentral.com/1471-2458/14/378/prepub
